# KAT2A: a prognostic biomarker influencing proliferation and immune escape in lung adenocarcinoma

**DOI:** 10.1186/s12885-025-15031-w

**Published:** 2025-11-12

**Authors:** Zhangmin Ke, Hao Xu, Kaikai Shen, Yuting Wen, Xia Pan, Zhenjue Qian, Li Wang, Suhua Zhu, Bing Wan, Yong Song

**Affiliations:** 1 Department of Respiratory and Critical Care Medicine, Jinling Hospital, Nanjing Medical University, Nanjing, 210002 China; 2 Department of Respiratory and Critical Care Medicine, Affiliated Jiangning Hospital of Nanjing Medicine University, Nanjing, 211100 China; 3 Department of Respiratory and Critical Care Medicine, The People’s Hospital of Danyang, Affiliated Danyang Hospital of Nantong University, Zhenjiang, 212300 China; 4 Department of Respiratory and Critical Care Medicine, Jinling Hospital, Affiliated Hospital of Medical School, Nanjing University, Nanjing, 210002 China; 5 Jiangning Clinical Medical School of Nanjing Medical University Kangda College, Nanjing, 211100 China

**Keywords:** Lung adenocarcinoma, KAT2A, Prognosis, Immune evasion

## Abstract

Lung adenocarcinoma (LUAD) is the most prevalent histological subtype of lung cancer, characterized by high mortality rates. KAT2A has been implicated in oncogenic processes and tumor progression. This study systematically investigated the role of KAT2A in LUAD through comprehensive analyses. Expression profiles and prognostic significance of KAT2A were evaluated using TCGA database and multiple GEO datasets. Functional enrichment analyses including GO and KEGG pathway analyses were conducted to elucidate biological mechanisms associated with KAT2A-regulated differentially expressed genes. Correlations between KAT2A expression levels and immune cell infiltration were analyzed using R software and publicly available databases. Experimental validation was performed through CCK-8 assays, colony formation assays, flow cytometry, and xenograft tumor models. Our findings demonstrated significantly elevated KAT2A expression in LUAD tissues and cells. Expression levels correlated with multiple clinicopathological parameters including TNM stage, pathological stage, sex, and tumor localization. High KAT2A expression was associated with reduced overall survival and exhibited prognostic relevance across diverse clinical subgroups. Multivariate analysis confirmed independent prognostic value of KAT2A expression in the established nomogram model. Functional annotation revealed enrichment of KAT2A-associated genes in critical biological processes and signaling pathways. Moreover, KAT2A expression exhibited correlations with mutational profiles and immune cell infiltration patterns in LUAD. Both in vitro and in vivo experiments demonstrated that KAT2A knockdown significantly suppressed tumor cell proliferation and immune evasion mechanisms, induced apoptosis, and inhibited tumor growth. These findings suggest that KAT2A may serve as a potential prognostic biomarker for LUAD, with therapeutic implications through its regulatory role in immune evasion pathways.

**Supplementary Information:**

The online version contains supplementary material available at 10.1186/s12885-025-15031-w.

## Introduction

Lung adenocarcinoma (LUAD) is the most prevalent histological subtype of lung cancer, constituting 40% of all lung cancer cases and 70% of non-small-cell lung cancer cases [1, 2]. Currently, LUAD exhibits the highest mortality rate among all malignancies [3]. Despite significant advances in therapeutic modalities, the clinical outcomes for LUAD remain dismal, with a 5-year overall survival rate below 20% [4, 5]. Multiple pathophysiological mechanisms, particularly immune escape, are recognized as critical determinants of poor prognosis. Immune evasion enables neoplastic cells to circumvent immune surveillance and programmed cell death, ultimately resulting in poor clinical outcomes [6].

KAT2A, also designated as GCN5, functions as a histone acetyltransferase and serves as a member of the GNAT family, regulating diverse biological processes including transcriptional regulation, cellular signaling, and inflammatory responses [7]. Recent research has highlighted the potential role of KAT2A in oncogenic transformation and tumor progression. Aberrant overexpression of KAT2A has been documented in multiple malignancies, where it correlates with aggressive tumor phenotypes and malignant biological behaviors [8]. In lung cancer specifically, KAT2A modulates key pathological processes such as endoplasmic reticulum stress and apoptotic pathways [9]. Notably, SF3B4-mediated regulation of KAT2A expression has been implicated in the initiation and development of LUAD [10]. However, the precise molecular mechanisms by which KAT2A contributes to LUAD pathogenesis, particularly its prognostic significance, remain incompletely characterized. Importantly, emerging evidence suggests that KAT2A participates in regulating both tumor cell proliferation and immune evasion in various cancers [11, 12]. Nevertheless, the specific regulatory roles of KAT2A in these critical processes within the context of LUAD have yet to be fully elucidated.

In this study, we systematically investigated the role of KAT2A in LUAD, with particular emphasis on its impact on patient prognosis, tumor progression mechanisms, and immune microenvironment dynamics. To comprehensively evaluate KAT2A’s clinical significance, we employed multi-omics data from public databases combined with R-based computational analyses to assess expression patterns, correlations with clinicopathological parameters, survival outcomes, and immune cell infiltration profiles. A robust nomogram model was developed to predict patient prognosis by integrating independent prognostic factors. The potential molecular mechanisms underlying KAT2A’s functions were further explored through functional enrichment analyses. Additionally, we experimentally validated the biological roles of KAT2A by examining its effects on LUAD cell line behavior and tumor growth in xenograft models. This research establishes a comprehensive framework for understanding KAT2A’s dual role as both a prognostic biomarker and a key regulator of LUAD progression, offering critical insights for future therapeutic strategies targeting immune evasion pathways.

## Materials and methods

### KAT2A expression prediction

The expression profiles of KAT2A across multiple cancer types and specifically in LUAD were systematically analyzed using the TIMER2.0 database (http://timer.cistrome.org/). LUAD-specific KAT2A expression data were further validated through multi-omics datasets from The Cancer Genome Atlas (TCGA) program (https://www.cancer.gov/ccg/research/genome-sequencing/tcga) and the Clinical Proteomic Tumor Analysis Consortium (CPTAC) database (https://proteomics.cancer.gov/programs/cptac). Transcriptomic datasets including GSE68571, GSE19188, GSE31210, and GSE63459 were retrieved from the Gene Expression Omnibus (GEO) database (https://www.ncbi.nlm.nih.gov/gds/) for comparative analysis. Statistical analyses were conducted using the ggplot2 package in R programming language. To investigate potential clinical correlations, LUAD patient clinical parameters were extracted from TCGA database and subjected to correlation analysis with KAT2A expression levels.

### Cell-type prediction

Single-cell transcriptomic profiles (GSE210347) were retrieved from the GEO database. Major cell clusters were identified using the R-based Monocle 3 package, and the results were visualized via Uniform Manifold Approximation and Projection (UMAP)-based dimensionality reduction.

### Cell culture

Human LUAD cell lines (A549, HCC827, PC-9, and H1975) and a normal human bronchial epithelial cell line (HBE) were obtained from in-house laboratory stocks. These cell lines were cultured in RPMI-1640 medium (Gibco, Grand Island, NY, USA) supplemented with 10% fetal bovine serum (FBS) and 1% penicillin/streptomycin, all sourced from the same supplier. Cells were incubated in a humidified atmosphere at 37 °C with 5% CO₂ under standard tissue culture conditions.

### Tissue sample collection

Six LUAD patients were recruited for this study. All participants were histologically confirmed to have LUAD and had no prior exposure to anticancer therapies prior to sample acquisition. Tumor and adjacent non-neoplastic tissues were collected during surgical resection procedures. The study protocol was approved by the Ethics Committee of the Affiliated Jiangning Hospital of Nanjing Medical University (approval number: 2024-03-135-K01). Written informed consent was obtained from each participant before tissue collection. The study was conducted in accordance with the principles outlined in the Declaration of Helsinki.

### Quantitative real-time polymerase chain reaction (qPCR)

Total RNA was isolated from clinical tissues and cell lines using TRIzol reagent (Invitrogen, Carlsbad, CA, USA). Following RNA extraction, 1 µg of RNA was reverse transcribed into first-strand cDNA using HiScript IV RT SuperMix for qPCR (with gDNA eraser; Vazyme, Nanjing, China). Quantitative PCR was performed using SYBR Green-based chemistry with the SupRealQ Purple Universal SYBR qPCR Master Mix (U+) (Vazyme) and first-strand cDNA as the template. β-actin was used as the internal control gene. Relative KAT2A expression levels were calculated using the 2^−ΔΔCT^ method.

### Western blot

Tissues and cells were lysed in Radioimmunoprecipitation Assay buffer to extract total proteins, and protein concentrations were quantified using the BCA protein assay kit (Beyotime, Shanghai, China). Equal amounts of protein were separated by SDS-PAGE and transferred onto PVDF membranes. After blocking with 5% non-fat milk, membranes were incubated with primary antibodies at 4 °C overnight and subsequently with secondary antibodies at room temperature for 2 h. Protein bands were detected using the SuperFemto ECL Master Mix (Vazyme).

### Immunohistochemistry (IHC)

KAT2A expression was evaluated using LUAD tissue microarrays. Tumor and adjacent non-neoplastic tissues within the microarrays were fixed in 10% neutral buffered formalin for 24 h, followed by paraffin embedding to create donor blocks. Tissue cores (1 mm in diameter) were punched from regions of interest (ROI) using an automated tissue arrayer (Chemicon International, Temecula, CA, USA) and transferred into recipient paraffin blocks. The blocks were incubated at 45 °C for 20 min to ensure complete integration of the tissue cores. Paraffin-embedded tissue-array Sect. (4 μm thickness) were prepared using a microtome. After routine dewaxing and rehydration, antigen retrieval was performed by heating in citrate buffer (pH 6.0). Endogenous peroxidase activity was inactivated using 3% hydrogen peroxide (H₂O₂), followed by blocking with normal goat serum. Sections were incubated with anti-KAT2A primary antibody at 4 °C overnight and subsequently with horseradish peroxidase (HRP)-conjugated secondary antibody at room temperature for 1 h. Immunohistochemical staining was visualized using a light microscope.

### Analysis of prognosis

Patient survival in LUAD was analyzed using the Kaplan-Meier plotter (https://www.kmplot.com/analysis/) and TCGA databases. Survival outcomes in patients with lung squamous cell carcinoma (LUSC) were evaluated using the Kaplan-Meier Plotter. Overall survival in patients with high versus low KAT2A expression was assessed using the survival R package.

### Nomogram model establishment and validation

Independent prognostic factors for LUAD were determined via univariate and multivariate Cox regression analyses. A nomogram model was developed using these prognostic factors through the survival and rms R packages. One-, three-, and five-year survival probabilities were calculated. The model’s predictive accuracy was validated by generating calibration curves to compare observed survival outcomes with estimated probabilities. The concordance index (C-index) was computed to evaluate model performance, with higher C-index values (approaching 1.0) reflecting enhanced predictive capability.

### Enrichment analysis

The LinkedOmics database (https://www.linkedomics.org/admin.php) was employed to identify genes associated with KAT2A in LUAD. Biological functions and pathways of these genes were systematically analyzed through Gene Ontology (GO) and Kyoto Encyclopedia of Genes and Genomes (KEGG) pathway enrichment analyses within the LinkedOmics platform. Additionally, Gene Set Enrichment Analysis (GSEA) was conducted using the ClusterProfiler R package, employing the c2.cp.all.v2022.1.Hs.symbols.gmt gene set as reference. Enrichment analysis results were visualized using the ggplot2 R package.

### Correlations of KAT2A expression with gene mutation and methylation

DNA methylation, mutation, and copy number variation (CNV) data were obtained from the TCGA database. Analyses conducted according to high- or low-KAT2A expression levels were performed utilizing the MethylMix and GenomeTornadoPlot R packages.

### Correlations of KAT2A expression with immune infiltration

Single-sample gene set enrichment analysis (ssGSEA), implemented in the Gene Set Variation Analysis (GSVA) R package, was applied to calculate immune cell enrichment scores. The association between KAT2A expression levels and immune infiltration was assessed using Pearson correlation analysis. Subsequently, correlations between KAT2A expression and infiltration levels of CD8 + T cells, CD4 + T cells (comprising regulatory T cells [Tregs] and conventional CD4 + T cells), as well as macrophages, were further investigated through the TIMER 2.0 database. Statistical comparisons of immune cell infiltration between high- and low-KAT2A expression groups were conducted using GSVA.

### Cell transfection

A549 and PC-9 cells were transfected with shRNA targeting KAT2A (shKAT2A) and a non-targeting control (shNC) using Lipofectamine 2000 transfection reagent (Invitrogen, Carlsbad, CA, USA) according to the manufacturer’s instructions.

### Cell counting kit-8 (CCK-8)

Transfected A549 and PC-9 cells cultured in 96-well plates were incubated at 37 °C for 0, 24, 48, and 72 h. Cell viability was determined using the CCK-8 assay (Yeasen, Shanghai, China). Specifically, 10 µL of CCK-8 reagent was added to each well, followed by a 4-hour incubation period. Optical density at 450 nm was measured using a microplate spectrophotometer (Bio-Rad, Hercules, CA, USA).

### Colony formation assay

A549 and PC-9 cells were cultured at 37 °C for 14 days. Following incubation, the cells were fixed with 4% paraformaldehyde and stained with crystal violet dye for 20 min. Colonies were visualized under a light microscope (Olympus) and quantified.

### Flow cytometry

Apoptosis was evaluated using the Annexin V–FITC/PI apoptosis detection kit (Yeasen, Shanghai, China). Briefly, A549 and PC-9 cells were resuspended in 100 µL of binding buffer provided in the kit. Subsequently, the cell suspension was incubated with 5 µL of Annexin V–FITC and 10 µL of PI for 10 min at room temperature (25 ± 2 °C). Following incubation, 100 µL of binding buffer was added to the mixture to dilute the stained cells, and apoptosis was quantified using a flow cytometer (Bio-Rad, Hercules, CA, USA) according to the manufacturer’s protocol.

### Cytotoxicity detection

Peripheral blood mononuclear cells (PBMCs), provided by Procell (Wuhan, China), were stimulated for 48 h with plate-bound anti-CD3e (1 µg/mL) and soluble anti-CD28 (0.5 µg/mL), both obtained from BioLegend (San Diego, CA, USA), in the presence of recombinant human IL-2 (50 IU/mL; PeproTech, Cranbury, NJ, USA). The pre-activated T cells (effector cells) were subsequently co-cultured with A549 or PC-9 target cells at an effector-to-target (E: T) ratio of 10:1.

Lactate dehydrogenase (LDH) cytotoxicity was quantified using the LDH cytotoxicity assay kit (Beyotime, Shanghai, China) after 24 h of co-culture. Supernatants were collected and analyzed according to the manufacturer’s protocol. Briefly, 100 µL of supernatant was incubated with 100 µL of LDH detection solution for 30 min under dark conditions. Optical density at 490 nm was measured using a microplate spectrophotometer (Bio-Rad, Hercules, CA, USA) to assess cytotoxic activity.

### Animal study

Male BALB/c nude mice (6–8 weeks old) were obtained from GemPharmatech Co., Ltd. (Nanjing, China). Animals were randomly assigned to two experimental groups (*n* = 6 per group). A549 cells stably transfected with shKAT2A or shNC were resuspended in PBS at a concentration of 1 × 10⁷ cells/mL. A total of 200 µL of the cell suspension was subcutaneously injected into the dorsal flank of each mouse. Tumor growth was monitored weekly using the formula: tumor volume = 0.5 × length × width² (mm³). After four weeks of tumor development, mice were euthanized via CO₂ inhalation. Tumors were harvested, photographed, and weighed for subsequent analysis.

### Immunofluorence

A549 cells were fixed with 4% paraformaldehyde for 15 min and permeabilized with 0.5% Triton X-100 for 20 min. Antigen retrieval was achieved by heating the samples in citrate buffer solution (pH 6.0) for 15 min. After blocking non-specific binding with 10% normal goat serum for 30 min at room temperature, the cells were incubated with anti-KAT2A primary antibody at 4 °C overnight. Subsequently, the cells were incubated with fluorescence-labeled secondary antibody at 37 °C for 1 h. Cell nuclei were stained with DAPI (1 µg/mL) for 5 min. Fluorescence images were captured using a confocal laser scanning microscope (Zeiss, Germany).

### Statistical analysis

The data were analyzed using GraphPad Prism 8, SPSS 25.0, and R 3.6.1. Group comparisons were conducted using Student’s t-test for normally distributed data or the Wilcoxon signed-rank test for non-normally distributed data. Correlation analysis was performed using Pearson’s correlation coefficient (for parametric data) or Spearman’s rank correlation coefficient (for non-parametric data). The diagnostic value of KAT2A was evaluated through receiver operating characteristic (ROC) curve analysis. Data are presented as mean ± standard deviation (SD), with statistical significance defined as *P* < 0.05.

## Results

### KAT2A is highly expressed in LUAD based on bioinformatics analysis

**Fig. 1:**
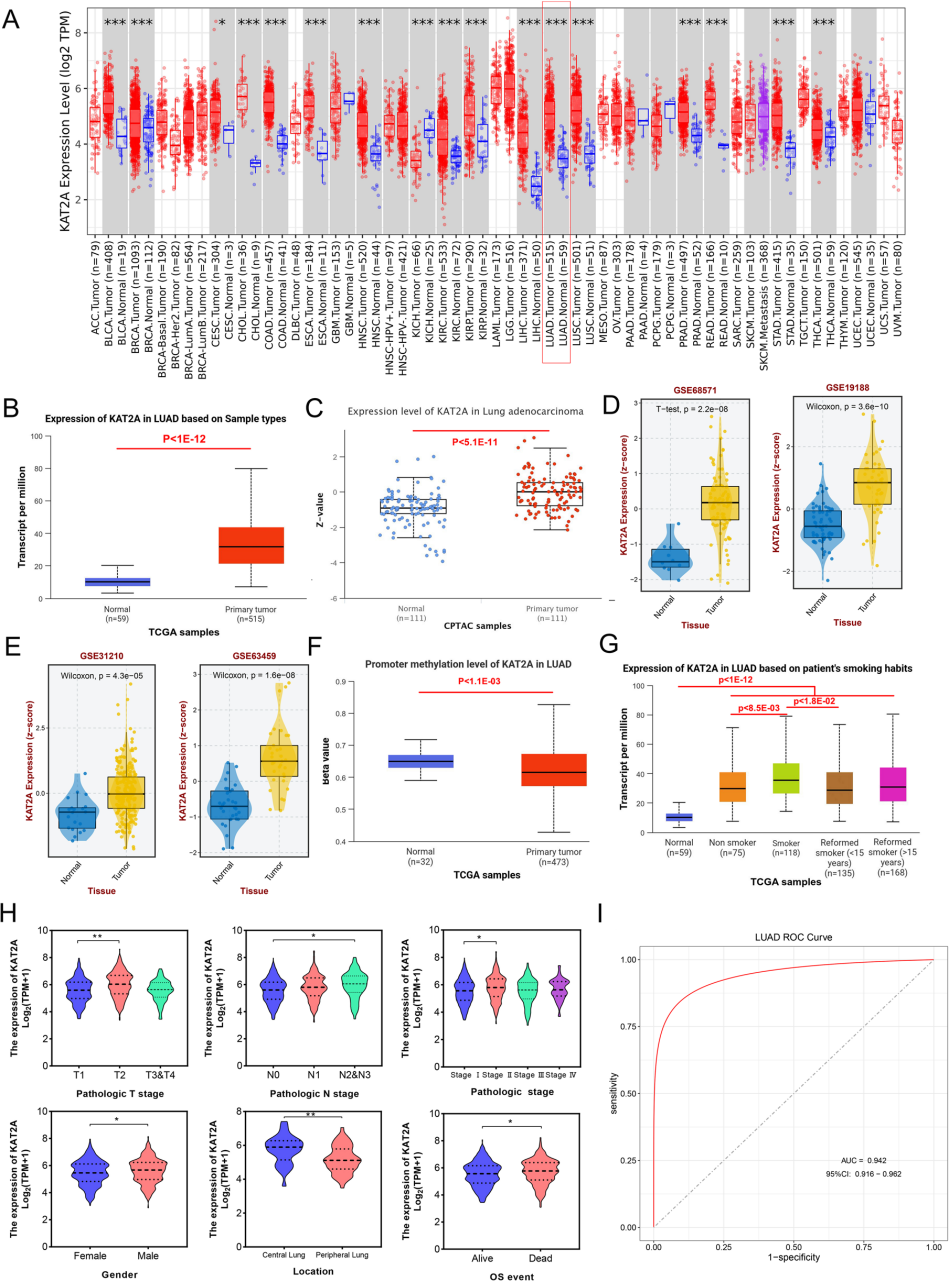
KAT2A is highly expressed in LUAD. **A** Pan-cancer analysis of KAT2A expression using the TIMER2.0 database. The red dots represent the tumor tissues, and the blue dots represent the adjacent normal tissues. The mRNA expression of KAT2A in LUAD tumor tissues and normal tissues was predicted using the (**B**) TCGA and (**C**) CPTAC databases. (**D**, **E**) KAT2A expression was predicted using the GSE68571, GSE19188, GSE31210, and GSE63459 datasets. **F** The promoter methylation level of KAT2A in LUAD was analyzed based on TCGA database. **G** The level of KAT2A in LUAD based on patients smoke habits was evaluated using TCGA database. **H** The level of KAT2A in LUAD from different T stage (T1, T2, T3&T4), *N* stage (N0, N1, N2&N3), pathologic stages (I, II, III, and IV), gender (female and male), tumor location (central and peripheral lung), and overall survival (alive and dead) was evaluated using the TCGA database. **I** The diagnostic value of KAT2A was analyzed using the ROC curve

We first examined KAT2A expression patterns across multiple cancer types using the TIMER2.0 database. As illustrated in Fig. 1A, KAT2A exhibited significantly higher expression in various tumors, including bladder urothelial carcinoma (BLCA), breast invasive carcinoma (BRCA), and other malignancies. Notably, its expression level in LUAD tumor tissues was markedly elevated compared to normal lung tissues. The differential expression of KAT2A in LUAD was further validated through integrative analysis of TCGA and CPTAC datasets. These multi-omics analyses demonstrated significantly increased KAT2A expression in primary tumor tissues relative to adjacent normal tissuess (Fig. 1B and C). To corroborate these findings, we conducted additional validation using independent GEO datasets (GSE68571, GSE19188, GSE31210, and GSE63459). Consistent with prior observations, all four datasets confirmed significantly upregulated KAT2A expression in tumor tissues versus normal controls (Fig. 1D and E). Epigenetic regulation of KAT2A was further investigated, revealing that promoter methylation levels were significantly reduced in LUAD tumor tissues compared to normal tissues (Fig. 1F). Subsequent analysis of smoking-related expression patterns showed that KAT2A expression was significantly higher in LUAD patients with a history of tobacco use compared to nonsmokers (Fig. 1G). Clinical relevance was further explored through TCGA-based analysis of KAT2A expression across different pathological stages. The results indicated significant upregulation in T2-stage tumors compared to T1-stage lesions. Similarly, N-stage progression correlated with increased KAT2A expression, with marked elevation observed in N2 and N3 stages relative to N0 stage. Additionally, KAT2A expression was significantly higher in stage II tumors compared to stage I, while no significant difference was detected between stages III/IV and stage I. When evaluating clinical features, we found distinct expression patterns associated with patient demographics and tumor characteristics. Specifically, KAT2A expression was significantly elevated in male patients compared to female counterparts. Interestingly, peripheral lung tumors exhibited notably lower KAT2A expression levels than central lung tumors, and deceased patients demonstrated significantly higher KAT2A expression compared to surviving patients (Fig. 1H). Finally, diagnostic potential assessment revealed that KAT2A expression yielded an AUC value of 0.942 in the TCGA cohort, indicating strong diagnostic value for LUAD detection (Fig. 1I).

### Single-cell atlas of patients with LUAD

**Fig. 2:**
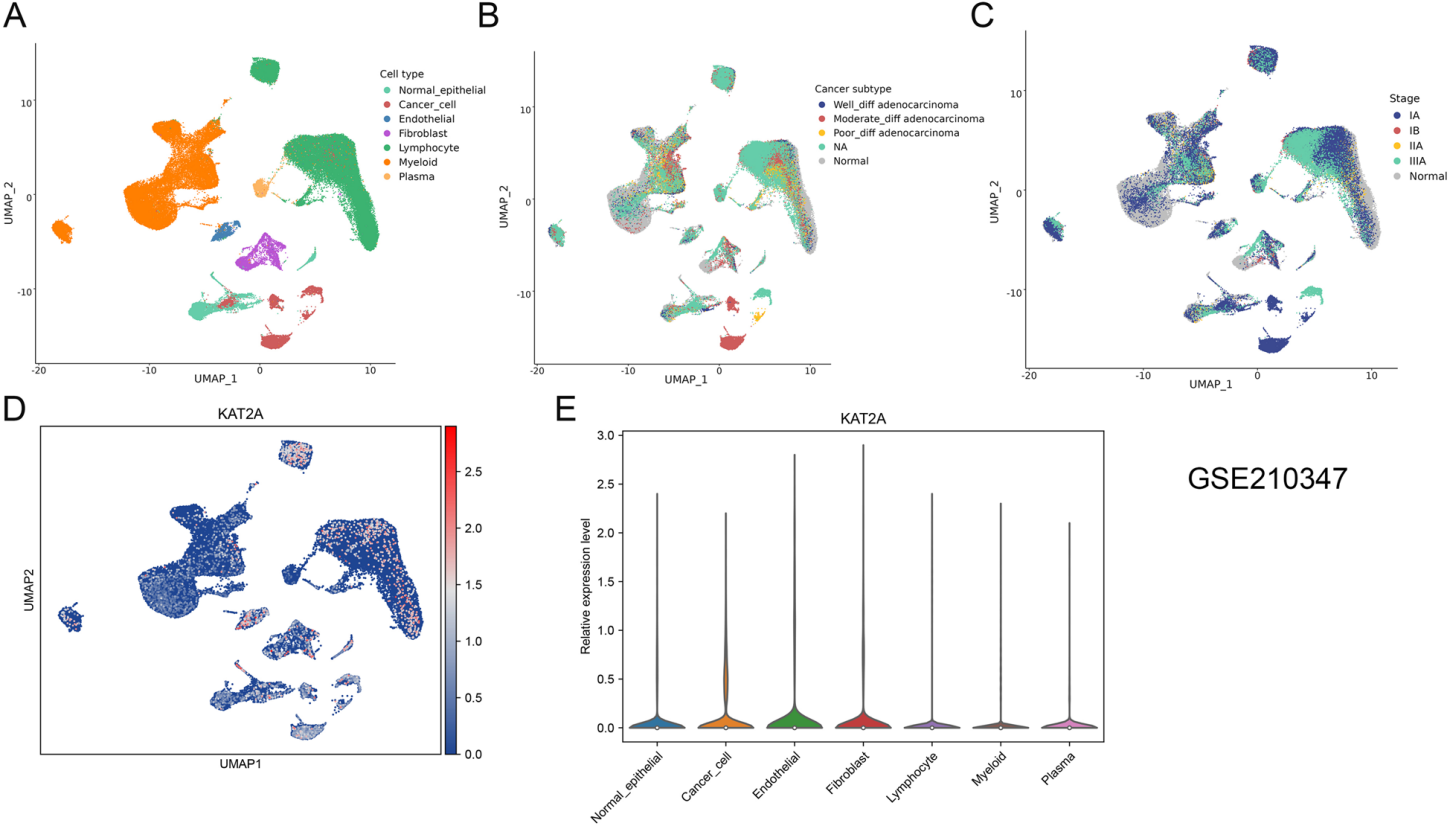
Single-cell atlas of patients with LUAD based on dataset GSE210347. **A** Cellular populations identified. **B** The UMAP plot shows cell clusters originating from either normal patients or LUAD patients, with different differentiation statuses. **C** The UMAP plot shows cell clusters originating from either normal patients or LUAD patients, categorized by different stages. **D** Specific expression patterns of KAT2A at the single-cell level. **E** The violin plot demonstrates the expression differences of the KAT2A gene across different cell types

Through analysis of the GSE210347 dataset, UMAP clustering revealed distinct partitioning of the LUAD tumor microenvironment into six major cellular components: cancer cells, endothelial cells, fibroblasts, lymphocytes, myeloid cells, and plasma cells, with clear morphological separation from normal epithelial cells (Fig. 2A). Further investigation demonstrated that cellular composition varied significantly among tumors with varying degrees of differentiation (well-differentiated, moderately differentiated, and poorly differentiated) as well as across pathological stages (IA, IB, IIA, and IIIA) (Fig. 2B and C). Notably, KAT2A expression was detected across all identified cell clusters, encompassing normal epithelial cells, cancer cells, endothelial cells, fibroblasts, lymphocytes, myeloid cells, and plasma cells (Fig. 2D and E).

### KAT2A is highly expressed in LUAD tissues and cell lines

**Fig. 3:**
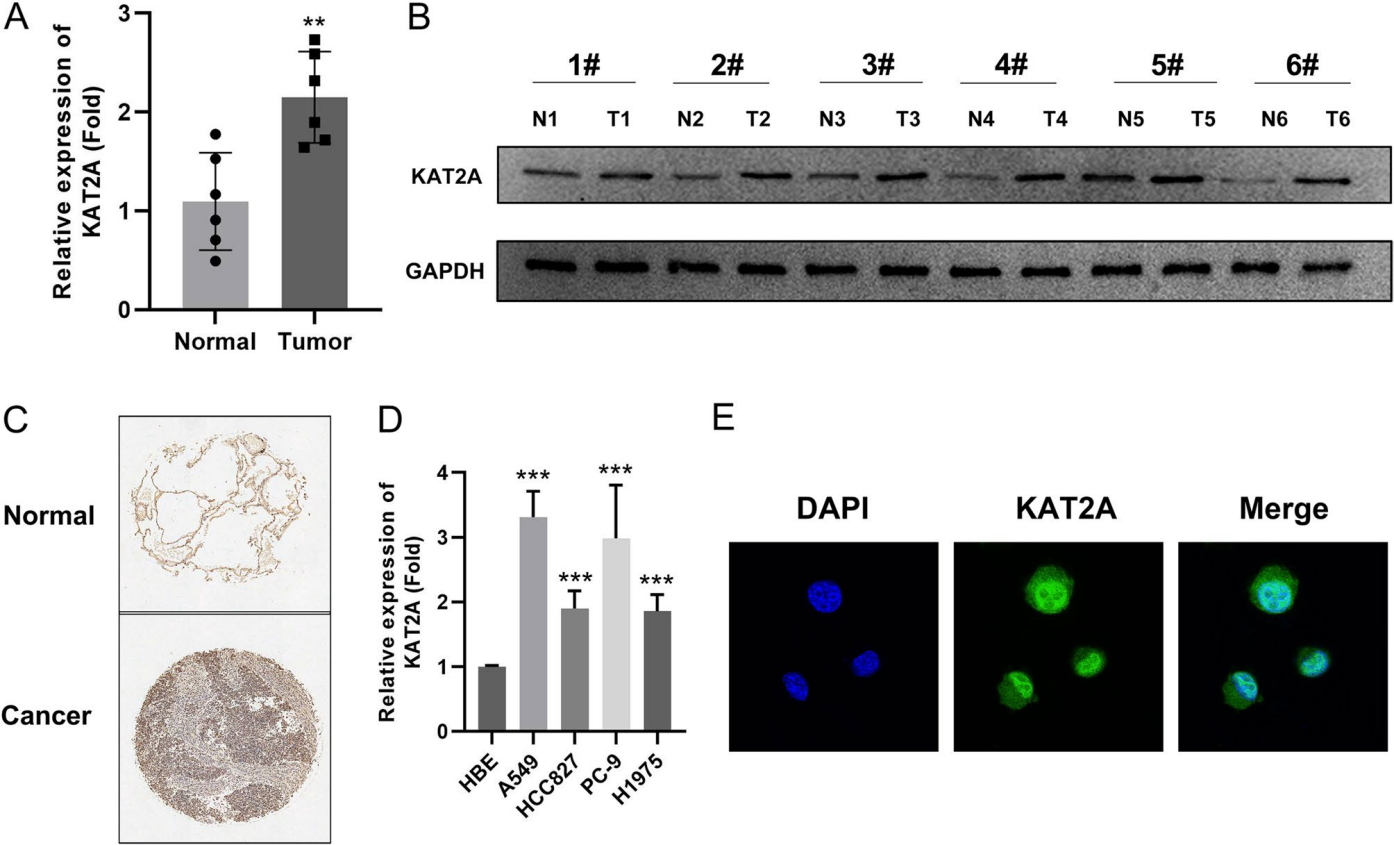
KAT2A is highly expressed in LUAD (**A**-**C**) The expression of KAT2A in tumor and adjacent normal tissues from patients with LUAD was detected using qPCR, western blot, and tissue microarray. **D** KAT2A expression in normal HBE cells and LUAD cell lines was detected using qPCR. **E** The location of KAT2A in LUAD cells were detected using immunofluorescence. **P < 0.01. ***P < 0.001

We collected tumor and adjacent normal tissues from LUAD patients and assessed KAT2A expression via qPCR, Western blot, and tissue microarray analyses. The results revealed that KAT2A was significantly upregulated in LUAD tumor tissues compared with adjacent normal tissues (Fig. 3A–C). To further validate this finding, KAT2A expression was examined in LUAD cell lines. qPCR analysis demonstrated that KAT2A expression was markedly elevated in LUAD-derived cell lines, including A549, HCC827, PC-9, and H1975 cells, relative to normal HBE cells (Fig. 3D). Notably, A549 and PC-9 cells exhibited the most pronounced upregulation of KAT2A, prompting their selection for subsequent functional studies. Additionally, we investigated the subcellular localization of KAT2A in LUAD cells and observed its presence in both the cytoplasm and nucleus, with significantly higher expression detected in the nucleus (Fig. 3E).

### High KAT2A expression is associated with poor prognosis of LUAD

**Fig. 4:**
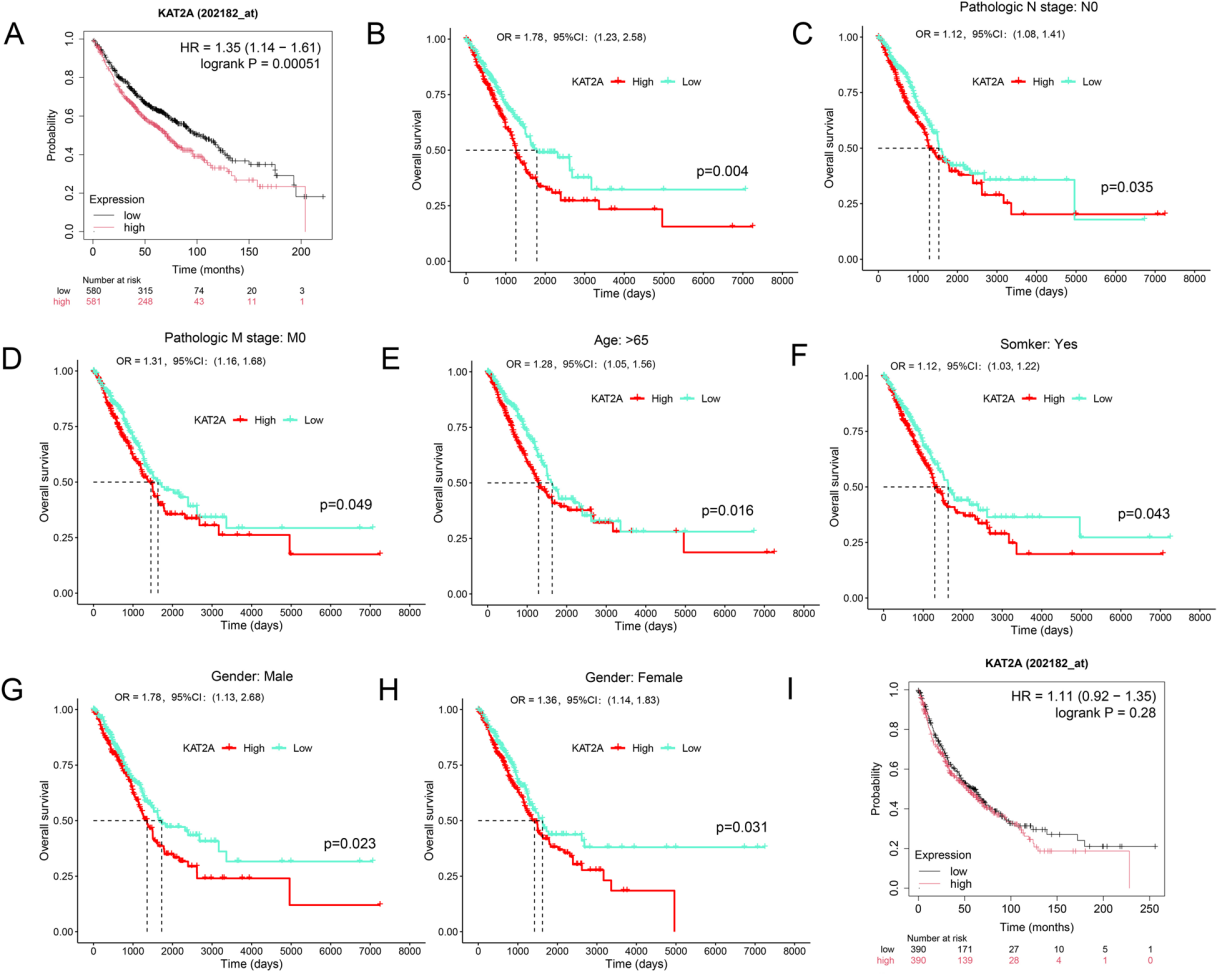
High KAT2A expression is associated with poor prognosis. The relationship between KAT2A expression and overall survival of patients was predicted using the (**A**) Kaplan-Meier plotter and (**B**) TCGA databases. The relationship between KAT2A expression and overall survival of patients in (**C**) N0 stage, (**D**) M0 stage, and (**E**) age > 65 years old was analyzed using TCGA database. The relationship between KAT2A expression and overall survival of (**F**) smokers, (**G**) males, and (**H**) females was also analyzed using TCGA database. **I** Prediction of overall survival with high or low KAT2A expression using the Kaplan-Meier plotter database

**Fig. 5:**
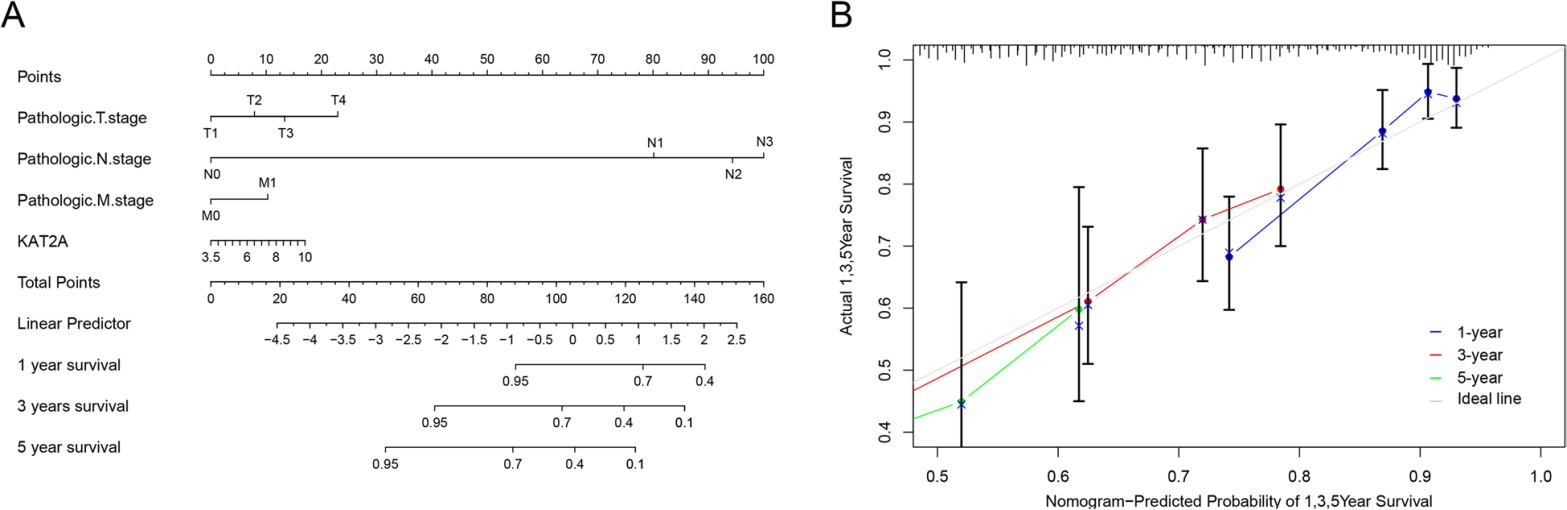
Nomogram model of LUAD. **A** A nomogram model was generated for predicting 1, 3, and 5-year overall survival. **B** The calibration curves for predicting 1, 3, and 5-year overall survival in the internal verification set

Based on prior evidence, we hypothesized that KAT2A expression correlates with overall survival. To validate this association, we analyzed prognostic outcomes in patients stratified by high or low KAT2A expression levels. Kaplan-Meier survival analysis combined with TCGA database interrogation revealed that LUAD patients with elevated KAT2A expression exhibited significantly reduced overall survival rates compared with those with low KAT2A expression (Fig. 4A–B). Subgroup analyses from TCGA clinical data further demonstrated that high KAT2A levels were associated with poorer survival outcomes in specific populations: N0-stage tumors, M0-stage tumors, patients aged > 65 years, smokers, and both male and female subgroups. At the median survival threshold, patients with high KAT2A expression consistently showed shorter survival durations than their low-expression counterparts (Fig. 4C–H). Notably, no significant survival differences were observed between high- and low-KAT2A expression groups in LUSC patients (Fig. 4I). Collectively, these data highlight the potential of KAT2A as a robust prognostic biomarker for LUAD. To enhance prognostic accuracy, we developed a nomogram integrating independent prognostic variables identified through multivariate Cox regression analysis. As detailed in Tables 1 and 2, clinicopathological parameters including T stage, N stage, M stage, and KAT2A expression were confirmed as significant independent predictors of LUAD prognosis. The nomogram model demonstrated strong predictive performance for 1-, 3-, and 5-year survival outcomes (Fig. 5A). Internal validation analyses confirmed high concordance between observed and predicted survival probabilities (Fig. 5B). Calibration curve assessment yielded a C-index of 0.753, indicating clinically meaningful predictive accuracy.

**Table 1:** LUAD risk prediction column chart assignment

Risk factors	Pathologic.T.stage				Pathologic.*N*.stage			
classification	T1	T2	T3	T4	N0	N1	N2	N3
score	0	7.5	12.5	25	0	80	94	100
Risk factors	Pathologic.M.stage				KAT2A			
classification	M0		M1		≤ 3.5		10	
score	0		15		0		20	

**Table 2:** LUAD risk prediction column chart scores and corresponding risk values

Total Points	88	124	142	
Linear Predictor	0.75	1	2	
1 year survival	0.95	0.7	0.4	
Total Points	64	100	118	136
Linear Predictor	2.2	0.25	0.75	1.65
3 year survival	0.95	0.7	0.4	0.1
Total Points	48	84	104	120
Linear Predictor	2.8	1	0	0.85
5 year survival	0.95	0.7	0.4	0.1

### Screening of KAT2A-related genes and enrichment analysis

**Fig. 6:**
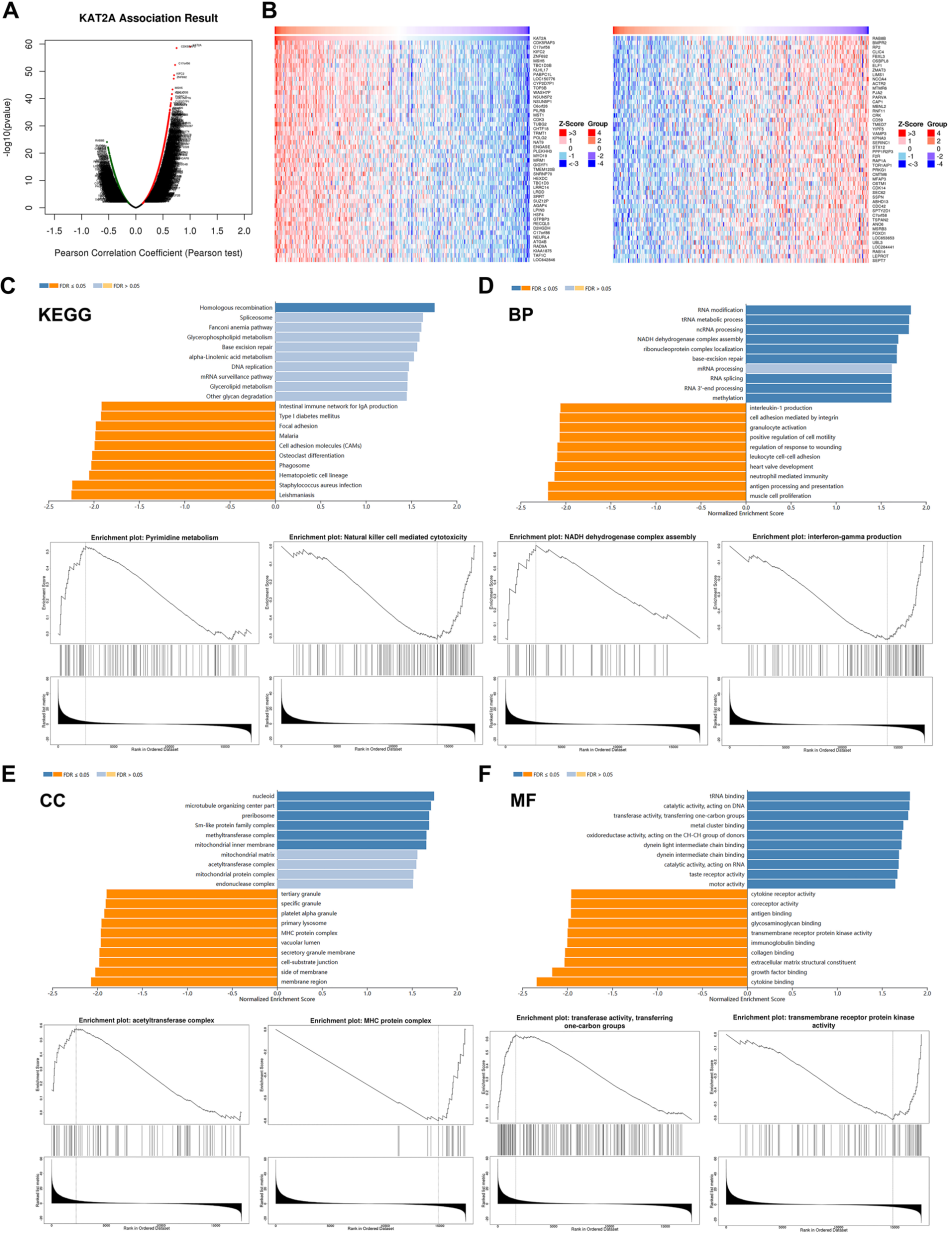
Screening of KAT2A-related genes and enrichment analysis. **A** Genes related to KAT2A expression in LUAD were obtained by LinkedOmics database. **B** The top 50 genes with positive and negative correlations of KAT2A. **C** The pathways involving these genes were predicted using KEGG enrichment analysis. (**D**-**F**) Biological processes (BP), cellular components (CC), and molecular functions (MF) of KAT2A-related genes were predicted using GO enrichment analysis

Next, we conducted a genome-wide analysis of KAT2A-associated genes in LUAD using the LinkedOmics database. This analysis revealed 612 genes with significant positive correlations to KAT2A, 684 genes with significant negative correlations, and 20,889 genes not significantly associated with KAT2A (Fig. 6A). The top 50 genes exhibiting the highest positive and negative correlations were visualized as heatmaps (Fig. 6B). To further characterize the functional implications of these associations, KEGG pathway and GO enrichment analyses were performed. KEGG pathway analysis demonstrated significant enrichment of KAT2A-associated genes across multiple pathways, with the top 10 positively and negatively correlated pathways illustrated in Fig. 6C. Notably, several of these pathways are directly implicated in oncogenic processes; for example, pyrimidine metabolism and natural killer cell-mediated cytotoxicity were among the most significantly associated pathways with LUAD progression. Through GO enrichment analysis, we systematically evaluated the biological functions of KAT2A-associated genes across three categories: biological processes (BP), cellular components (CC), and molecular functions (MF). In the BP category, NADH dehydrogenase complex assembly and interferon-gamma production exhibited the strongest correlations with LUAD (Fig. 6D). For the CC category, the acetyltransferase complex and MHC protein complex showed the most significant associations with LUAD (Fig. 6E). In the MF category, transferase activity involving one-carbon group transfer and transmembrane receptor protein kinase activity demonstrated the highest relevance to LUAD pathogenesis (Fig. 6F). Collectively, these findings highlight the multifaceted role of KAT2A in LUAD, involving extensive interactions with diverse genes and pathways critical to tumor biology.

### KAT2A is related to immune characteristics

**Fig. 7:**
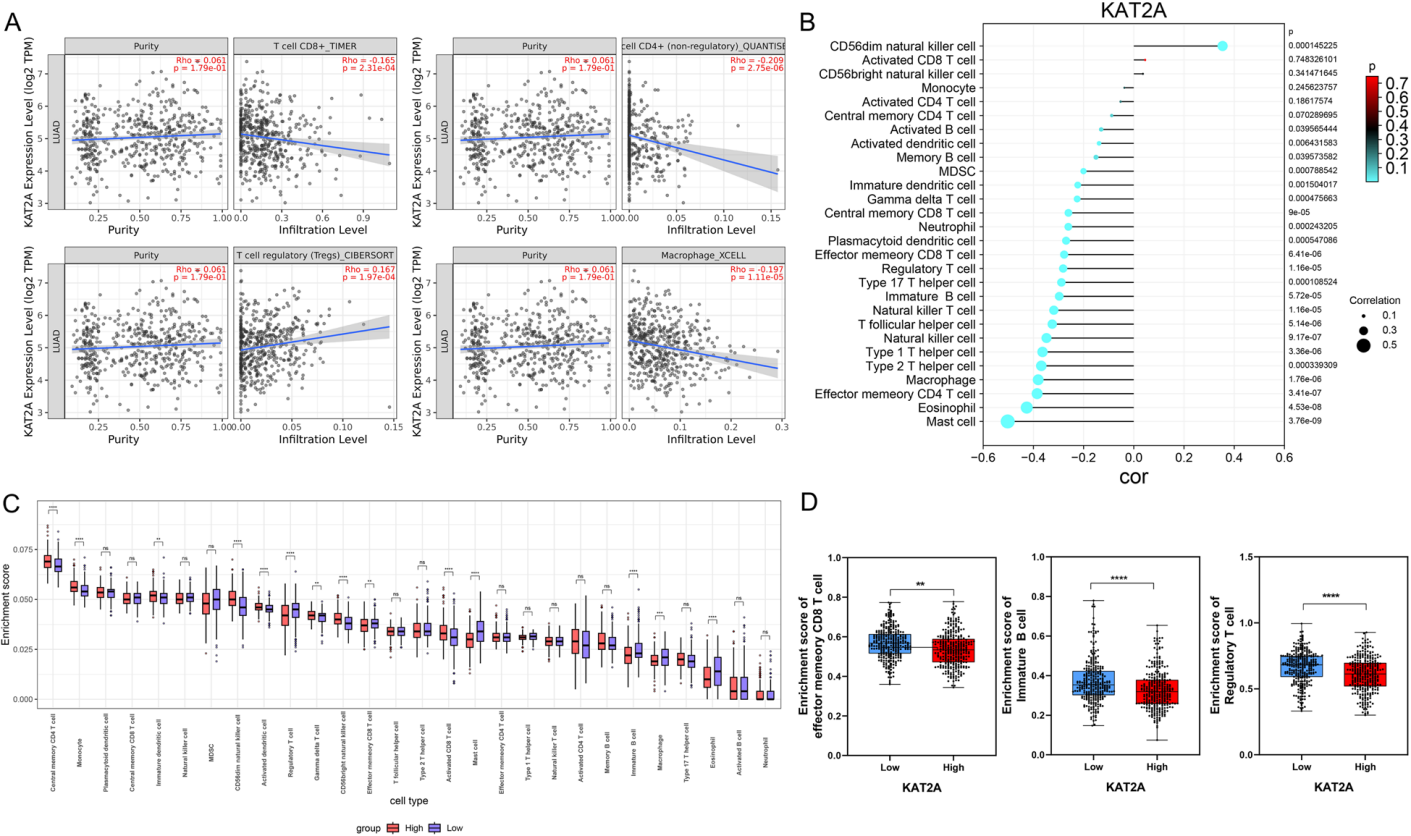
KAT2A is related to immune characteristics. **A** The correlation between KAT2A expression and immune cells including CD8 + T cells, CD4 + T (non-regulatory) cells, Treg cells, and macrophages using the TIMER2.0 database. **B** The correlation between KAT2A and immune infiltrating cells in LUAD. **C** Comparation of immune infiltrating cells in the KAT2A high and low groups. Infiltration levels of (**D**) effector memeory CD8 + T cells, B cells, and regulatory T cells in the KAT2A high and low groups. **P < 0.01. ***P < 0.001. ****P < 0.0001. ns: no significance

Immune evasion represents a pivotal mechanism driving the progression of LUAD. To investigate this relationship, we analyzed the correlation between KAT2A expression and immune cell infiltration. The results demonstrated that KAT2A expression showed significant positive correlations with regulatory T (Treg) cells and exhibited marked negative correlations with CD8 + T cells, CD4 + T cells (non-regulatory), and macrophages (Fig. 7A). Furthermore, KAT2A expression was positively associated with CD56dim natural killer cells while displaying negative correlations with multiple immune infiltration cell types, including mast cells, eosinophils, CD4 + T cells, macrophages, and T helper cells (Fig. 7B). Stratification of KAT2A expression into high- and low-expression groups revealed distinct immune infiltration profiles. Notably, several immune cell types—central memory CD4 + T cells, monocytes, CD56dim NK cells, activated dendritic cells, and regulatory T cells—showed significant differences in abundance between these groups (Fig. 7C). Of particular importance, effector memory CD8 + T cells, B cells, and regulatory T cells were significantly reduced in the high-KAT2A expression group compared with the low-KAT2A group (Fig. 7D). These findings suggest that KAT2A may modulate immune evasion in LUAD by altering the tumor microenvironment’s immune cell composition.

### KAT2A expression affects gene mutation and DNA methylation

**Fig. 8:**
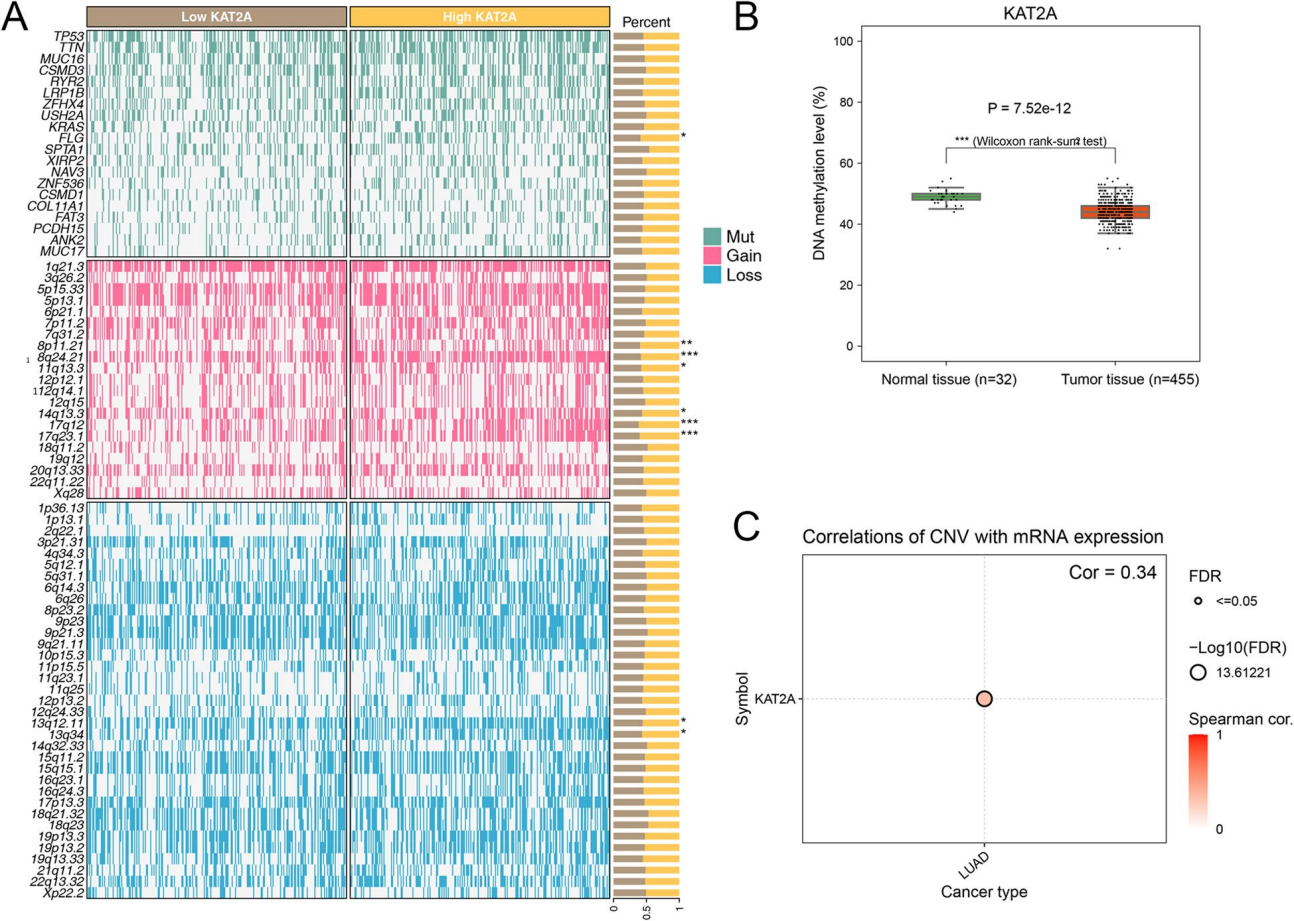
Relationship between KAT2A expression and gene mutations, DNA methylation, and CNV. **A** The heatmap shows the proportion differences of mutated genes (upper part of the figure), gene copy number gain (middle part of the figure), and loss (lower part of the figure) in tumor tissues with high and low KAT2A gene expression. **B** The boxplot illustrates the differences in DNA methylation between tumor tissues and normal tissues. **C** The bubble plot displays the correlation between KAT2A gene expression and CNV in LUAD tumor tissues. *R* = 0.34

Mutant genes associated with high- and low-KAT2A expression were visualized using a heatmap. The analysis revealed significant differences in gene mutation profiles, copy number gains, and losses between the high- and low-KAT2A expression groups (Fig. 8A). Additionally, DNA methylation levels were demonstrated to be significantly reduced in tumor tissues compared with normal tissues (Fig. 8B). To further investigate the relationship between KAT2A and CNV, we conducted Spearman’s rank correlation analysis. The results indicated a moderate positive correlation between KAT2A expression and CNV in LUAD (*R* = 0.34; Fig. 8C).

### Knockdown of KAT2A inhibits the progression of LUAD

**Fig. 9:**
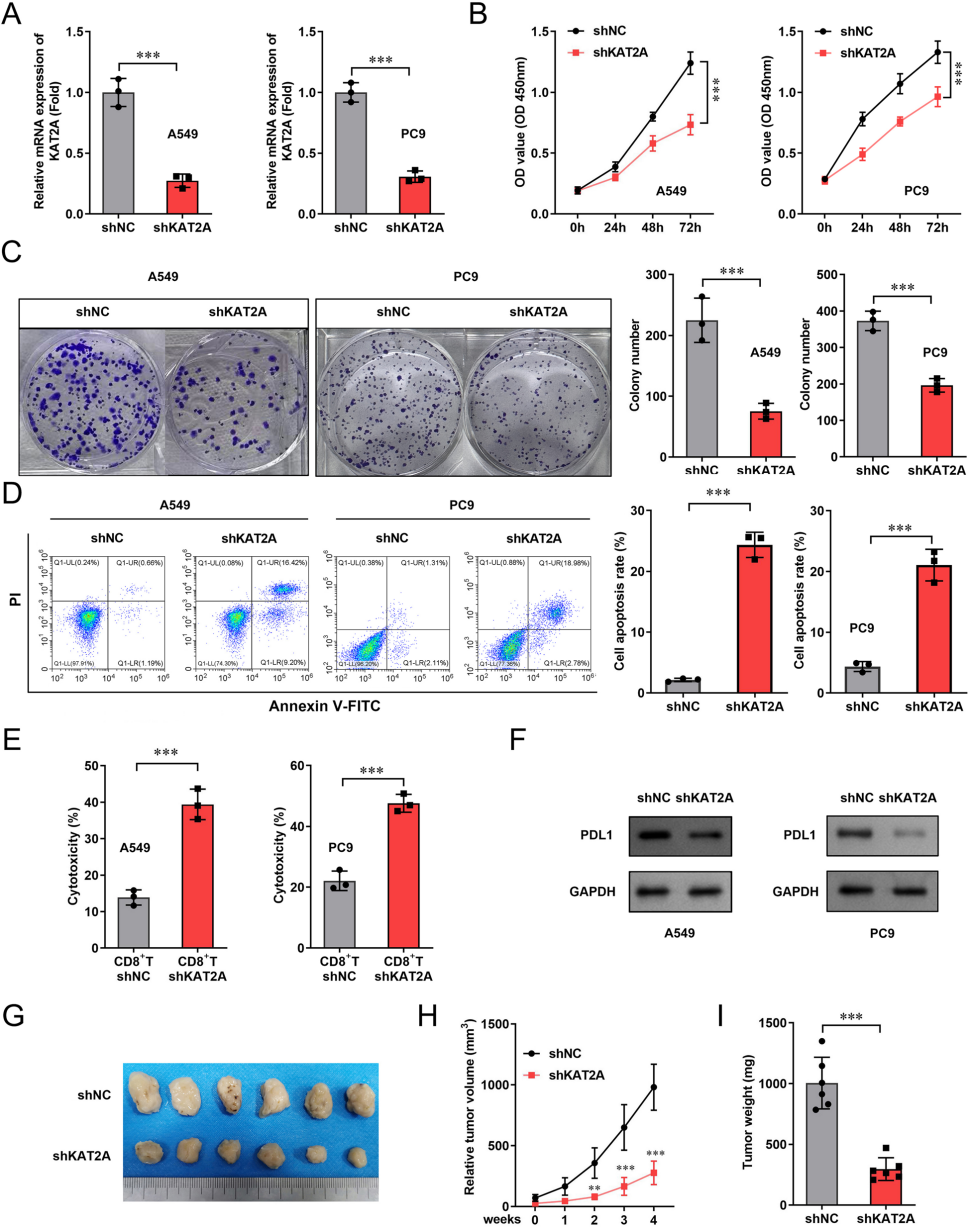
Knockdown of KAT2A inhibits the progression of LUAD. A549 and PC-9 cells were transfected with shNC and shKAT2A, and (**A**) transfection efficiency was detected by qPCR. (**B**) cell viability was detected using the CCK-8. **C** Colony formation assay was conducted to assess cell proliferation. **D** Flow cytometry was used to evaluate cell apoptosis. **E** Cytotoxicity to tumor cells was detected by the LDH assay kit. **F** PD-L1 protein levels were measured by western blot. (**G**-**H**) Images of tumors isolated from xenograft tumor model mice, and tumor volume was measured every week after model generation. **I** Tumor weight was detected. **P < 0.01. ***P < 0.001

The role of KAT2A in LUAD progression was investigated using in vitro and in vivo experimental models. To assess its functional impact, we evaluated cell viability, apoptosis, and immune evasion following KAT2A knockdown. First, shRNA targeting KAT2A was transfected into A549 and PC-9 cells, which effectively reduced KAT2A mRNA expression levels (Fig. 9A). The results revealed that KAT2A knockdown significantly suppressed cell viability and colony-forming capacity (Fig. 9B and C). In contrast, KAT2A silencing markedly increased apoptotic cell death (Fig. 9D). Furthermore, CD8 + T cells exhibited enhanced cytotoxic activity against tumor cells in response to KAT2A depletion (Fig. 9E). Notably, PD-L1 expression in LUAD cells was significantly downregulated following KAT2A knockdown (Fig. 9F). To validate these findings in vivo, a xenograft tumor model was established. Tumor size, volume, and weight were significantly reduced in mice with KAT2A knockdown compared with control groups (Fig. 9G-I).

## Discussion

In the present study, we investigated the role of KAT2A in LUAD. As demonstrated by pan-cancer analysis, KAT2A expression is significantly upregulated in multiple cancer types [13]. The dysregulation of gene expression between tumor and adjacent normal tissues represents a fundamental mechanism underlying tumor initiation and progression [14]. Consequently, KAT2A functions as a critical regulator in oncogenesis and malignant transformation [15]. In colorectal cancer, KAT2A overexpression has been shown to enhance cell proliferation, migration, invasion capabilities, and glycolytic metabolism [11]. Furthermore, functional studies in hepatocellular carcinoma reveal KAT2A’s involvement in suppressing tumor cell proliferation, inducing G1 phase cell cycle arrest, and promoting apoptotic pathways [16]. Additional evidence from pancreatic cancer models demonstrates that KAT2A facilitates malignant progression through mechanisms involving enhanced proliferation, migratory capacity, invasive potential, epithelial-mesenchymal transition, and metabolic reprogramming [17]. However, the specific molecular role of KAT2A in LUAD remains poorly characterized. A single prior investigation reported that SF3B4 drives LUAD progression via modulation of KAT2A 5’ alternative splicing [10]. This knowledge gap necessitated further mechanistic exploration of KAT2A’s tumor-promoting functions in LUAD. Our current findings demonstrate that KAT2A expression is consistently elevated across various malignancies including LUAD, corroborating previous reports [18]. These observations were validated in both LUAD cell culture systems and clinical tissue specimens. Notably, ROC curve analysis revealed robust diagnostic performance of KAT2A for LUAD detection, suggesting its potential as a clinical biomarker. This diagnostic utility parallels previously documented findings demonstrating KAT2A’s value as a diagnostic indicator in hepatocellular carcinoma [19].

In the subsequent analysis, we examined the association between KAT2A expression levels and clinical parameters in LUAD patients. As established in prior studies, multiple risk factors contribute to LUAD pathogenesis, including tobacco exposure, chronological aging, sex distribution, and dietary patterns [20]. Our findings revealed that KAT2A expression exhibited significant differences between T1/T2 stages, N0 vs. N2/N3 classifications, and pathological stages I/II according to the TNM staging system. These observations indicate a strong correlation between KAT2A expression profiles and TNM-based clinical staging. Furthermore, statistical analyses demonstrated significant associations between KAT2A expression and patient demographics (sex), tumor localization, and overall survival rates, suggesting its potential role as a prognostic indicator. These correlations implicate KAT2A in both tumor progression mechanisms and clinical outcome prediction for LUAD patients.

We investigated the impact of KAT2A expression on prognostic outcomes in LUAD. Prognostic prediction is critical for tailoring clinical interventions. Despite the identification of numerous candidate prognostic biomarkers, few have achieved widespread clinical adoption. Thus, there remains an urgent need for reliable prognostic indicators. Emerging evidence supports KAT2A as a potential prognostic marker in malignancies. Yu et al. [21] demonstrated that elevated KAT2A expression correlates with immune microenvironment infiltration, malignant progression, and reduced survival rates in diffuse large B-cell lymphoma. In pancreatic cancer, high KAT2A levels have been linked to adverse clinical outcomes [22]. Similarly, Vitkevičienė et al. [23] reported that increased KAT2A expression serves as an independent predictor of poor survival in acute myeloid leukemia patients. In our study, high KAT2A expression significantly correlated with decreased overall survival in LUAD patients compared to low-expression cohorts. Furthermore, elevated KAT2A levels were associated with poor prognosis in subgroups defined by N0 stage, M0 stage, age > 65 years, smoking history, and across both sexes. Notably, this prognostic association was not observed in LUSC patients, underscoring LUAD-specific relevance. These data suggest that KAT2A expression holds promise as a prognostic biomarker, particularly for LUAD patients with distinct clinical characteristics. To integrate KAT2A into clinical practice, we evaluated its utility within established prognostic frameworks. A nomogram, a quantitative tool for individualized cancer prognosis prediction [24], was employed alongside the TNM staging system [25], the current standard for LUAD risk stratification. Our analysis revealed that the N stage emerged as the most influential independent prognostic factor, while T stage, M stage, and KAT2A expression also contributed to prognostic accuracy. This hierarchical contribution highlights the complementary role of KAT2A in enhancing LUAD prognosis prediction beyond conventional staging metrics.

Multiple genes correlated with KAT2A expression were also identified. Further enrichment analysis revealed that KAT2A is involved in various biological functions and signaling pathways. KEGG analysis indicated that KAT2A expression was negatively associated with natural killer cell-mediated cytotoxicity, suggesting a role for KAT2A in promoting immune evasion. This is because natural killer cells play a critical role in innate immune surveillance; the stronger their cytotoxic activity against tumor cells, the more difficult it becomes for tumors to evade immune detection [26]. Moreover, KAT2A expression was negatively correlated with interferon-gamma production and MHC protein complex expression, both of which are essential for effective antitumor immunity [27, 28]. In addition, pyrimidine metabolism is a hallmark of cancer that supports aberrant tumor proliferation and contributes to epithelial-mesenchymal transition and chemotherapy resistance [29]. The acetyltransferase complex is also known to regulate cancer cell proliferation [30]. Our data showed that KAT2A expression was positively correlated with both pyrimidine metabolism and the acetyltransferase complex, suggesting that KAT2A may influence tumor proliferation.

A relationship between KAT2A expression and immune cell infiltration was also predicted. Immune evasion often leads to malignant progression, metastasis, and poor prognosis, representing one of the major challenges in cancer therapy [31]. KAT2A has been shown to interact with ACSS2 to promote brain tumor growth and immune evasion [12]. In this study, we found that KAT2A expression was negatively associated with CD8 + T cells, macrophages, and CD4 + T (non-regulatory) cells, while it was positively correlated with regulatory T cells (Tregs). Multiple immune cell types play pivotal roles in tumor immune evasion. CD8 + T cells exert direct anti-tumor effects through cytotoxicity, whereas CD4 + T cells, as central mediators of both innate and adaptive immunity, also contribute to anti-tumor responses [32, 33]. Moreover, macrophage-driven inflammation can facilitate tumor immune escape [34]. Although our findings indicate that KAT2A expression is closely linked to immune evasion in LUAD, its associations with different immune-infiltrating cell populations are distinct. The positive correlations between KAT2A expression and central memory CD4 + T cells (Tcm), activated CD8 + T cells, and activated dendritic cells (aDCs) suggest that KAT2A may enhance adaptive immune responses by promoting T cell memory maintenance and improving antigen presentation efficiency. Conversely, the negative associations with Tregs and macrophages imply that KAT2A might suppress the infiltration of immunosuppressive cell types through epigenetic regulation. This highlights the highly complex regulatory network within the tumor microenvironment. Extensive experimental validation is therefore required to fully elucidate the impact of KAT2A on the tumor immune landscape.

Thus, DNA methylation may serve as a diagnostic biomarker and a potential therapeutic target in lung cancer [35]. We found that the promoter methylation levels of KAT2A were significantly lower in LUAD tissues, providing new insights into the regulatory mechanisms underlying KAT2A expression in LUAD.

Using bioinformatics analysis, we explored the potential role of KAT2A in the progression of LUAD. However, the exact role of KAT2A in LUAD development remains to be confirmed through experimental validation. Therefore, we conducted both in vitro and in vivo experiments to verify the biological functions of KAT2A in LUAD. The results demonstrated that KAT2A silencing suppressed cell proliferation and immune evasion, promoted apoptosis of LUAD cells, and inhibited tumor growth in xenograft mouse models. These findings indicate that KAT2A functions as an oncogene in LUAD, suggesting its potential as a therapeutic target. Notably, KAT2A knockdown led to reduced PD-L1 protein expression in LUAD cells, implying a potential role for KAT2A in regulating the expression of immune checkpoint molecules. As a histone acetyltransferase, KAT2A catalyzes the acetylation of lysine residues on histones, resulting in chromatin relaxation and enhanced gene transcription [36]. Given that PD-L1 expression is regulated by complex transcriptional and post-translational mechanisms—including glycosylation, ubiquitination, phosphorylation, and acetylation—across various cancers [37], it is plausible that KAT2A may promote PD-L1 transcription by modulating histone acetylation at its promoter or enhancer regions. This hypothesis is supported by previous studies showing that histone acetylation plays a critical role in regulating PD-L1 expression in tumor cells [38]. For example, inhibition of histone deacetylases (HDACs) has been shown to upregulate PD-L1 expression in cancer [39]. Moreover, KAT2A has been identified as a lactyltransferase that catalyzes histone H3 lactylation, thereby promoting PD-L1 expression and driving tumor growth and immune evasion in brain tumors [12]. Therefore, as a key histone acetyltransferase, KAT2A may establish an open chromatin configuration at the PD-L1 locus, facilitating its transcription and contributing to immune evasion. However, the precise molecular mechanism by which KAT2A regulates PD-L1—such as whether it directly binds to the PD-L1 promoter or acts through intermediary transcription factors—requires further investigation.

Although our study provides a comprehensive analysis of the potential role of KAT2A in LUAD, it is important to acknowledge certain limitations.

First, bioinformatics analysis relies heavily on public databases such as TCGA and GEO, and integrating data from multiple cohorts may introduce batch effects or platform-specific biases. Our clinical sample size was relatively small, and future studies should expand the cohort to further validate the clinical relevance of KAT2A expression. Additionally, Our in vivo experiments assessed tumor growth using nude mice, which lack functional T cells, and therefore did not allow evaluation of the tumor immune microenvironment. No experimental validation was performed for the bioinformatically predicted associations with immune infiltration, and this will be addressed in future work using immunocompetent mouse models. Finally, our study did not examine the potential heterogeneity of KAT2A’s role across molecular subtypes of LUAD (such as EGFR-mutant versus KRAS-mutant tumors), which could influence its functional impact and therapeutic implications.

Clinically, the development of selective KAT2A inhibitors, either as monotherapy or in combination with anti-PD-1/PD-L1 antibodies, could be explored in LUAD patients with high KAT2A expression. Such a strategy may enhance response rates and help overcome primary or acquired resistance to ICB. Furthermore, given that KAT2A expression correlates with aggressive clinicopathological features, it has the potential to serve as a predictive biomarker for patient stratification in future clinical trials. Further preclinical studies using immunocompetent mouse models and patient-derived organoids are warranted to validate the efficacy and safety of this combinatorial approach and to determine the optimal timing and dosing regimens for translational applications.

In conclusion, we found that KAT2A is highly expressed in LUAD, suggesting that KAT2A may serve as a promising prognostic biomarker for patients with LUAD. Moreover, KAT2A was associated with immune evasion, and its knockdown was shown to decelerate LUAD progression. These findings provide new insights into the role of KAT2A and its underlying molecular mechanisms, and offer a theoretical foundation for its potential use as a diagnostic and prognostic marker as well as a therapeutic target in LUAD.

## Supplementary Information

Supplementary Material 1.

## Data Availability

The datasets used and/or analyzed during the current study are available from the corresponding author on reasonable request.
