# Supplementary figures and images for: KAT2A: a prognostic biomarker influencing proliferation and immune escape in lung adenocarcinoma
Source: BMC Cancer. 2025 Nov 12;25:1753. doi: 10.1186/s12885-025-15031-w (PMC12613538; doi:10.1186/s12885-025-15031-w)

Figure 3B


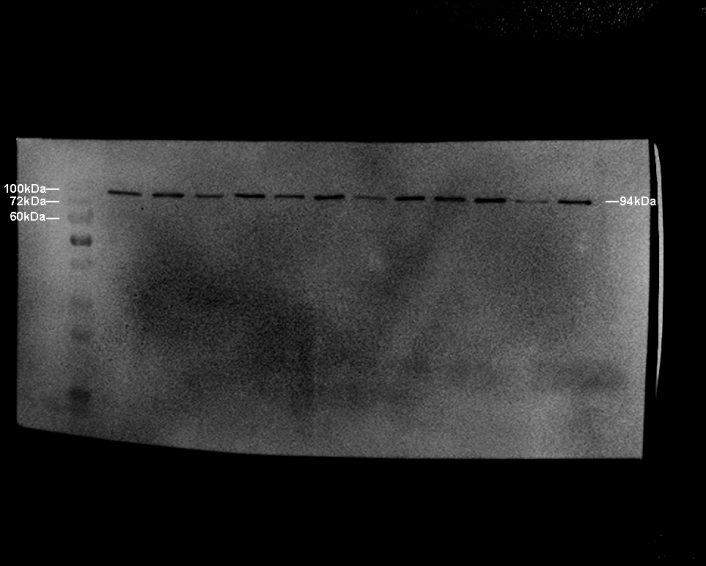


1. KAT2A


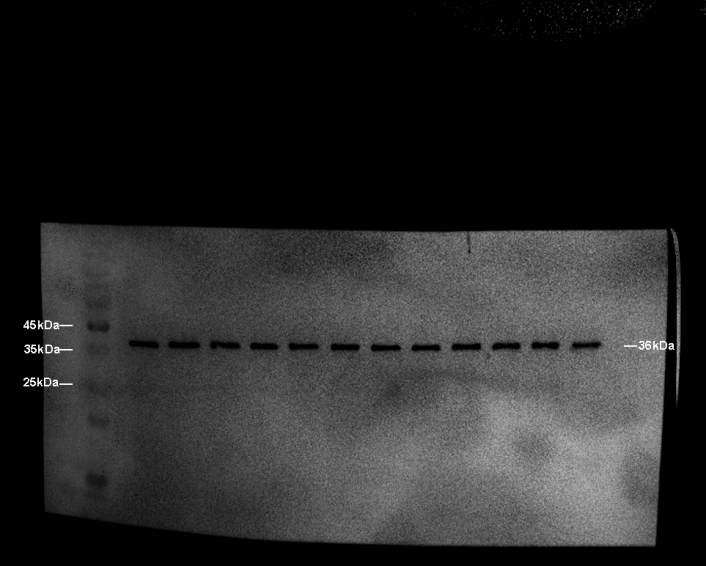


1. GAPDH

Figure 9E


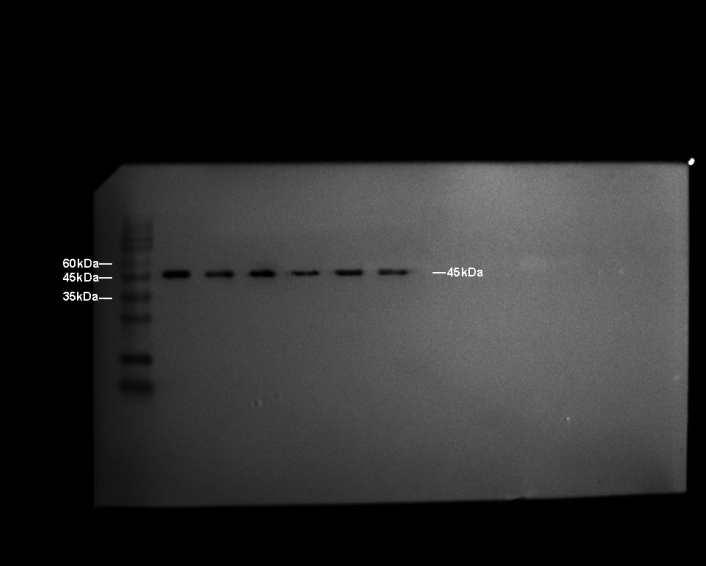


1. PD-L1


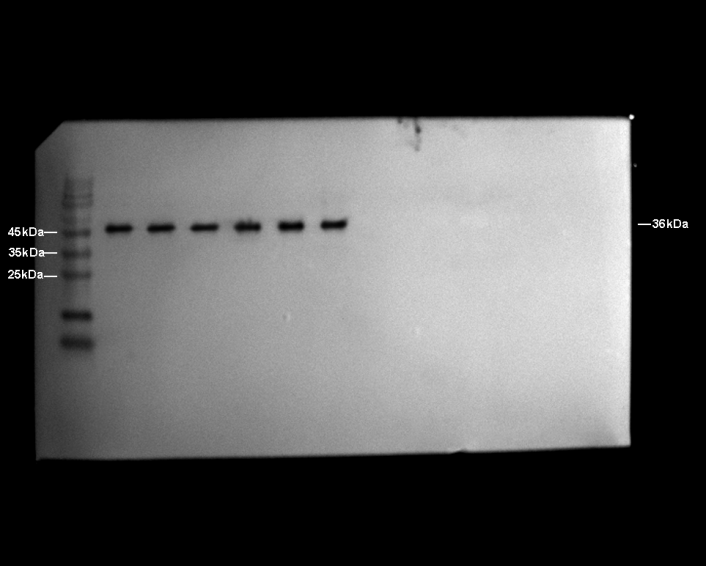


2-GAPDH

Supplement: Supplementary file 1 — Supplementary Material 1. [file 12885_2025_15031_MOESM1_ESM.docx]
